# Supplementary material for: Using smartphone step counts to monitor patients with total hip arthroplasty: The impact of patients’ living arrangements and residential location
Source: PLoS One. 2025 Jun 27;20(6):e0326338. doi: 10.1371/journal.pone.0326338 (PMC12204548; doi:10.1371/journal.pone.0326338)
Supplement: S1 Fig — (DOCX) [file pone.0326338.s001.docx]

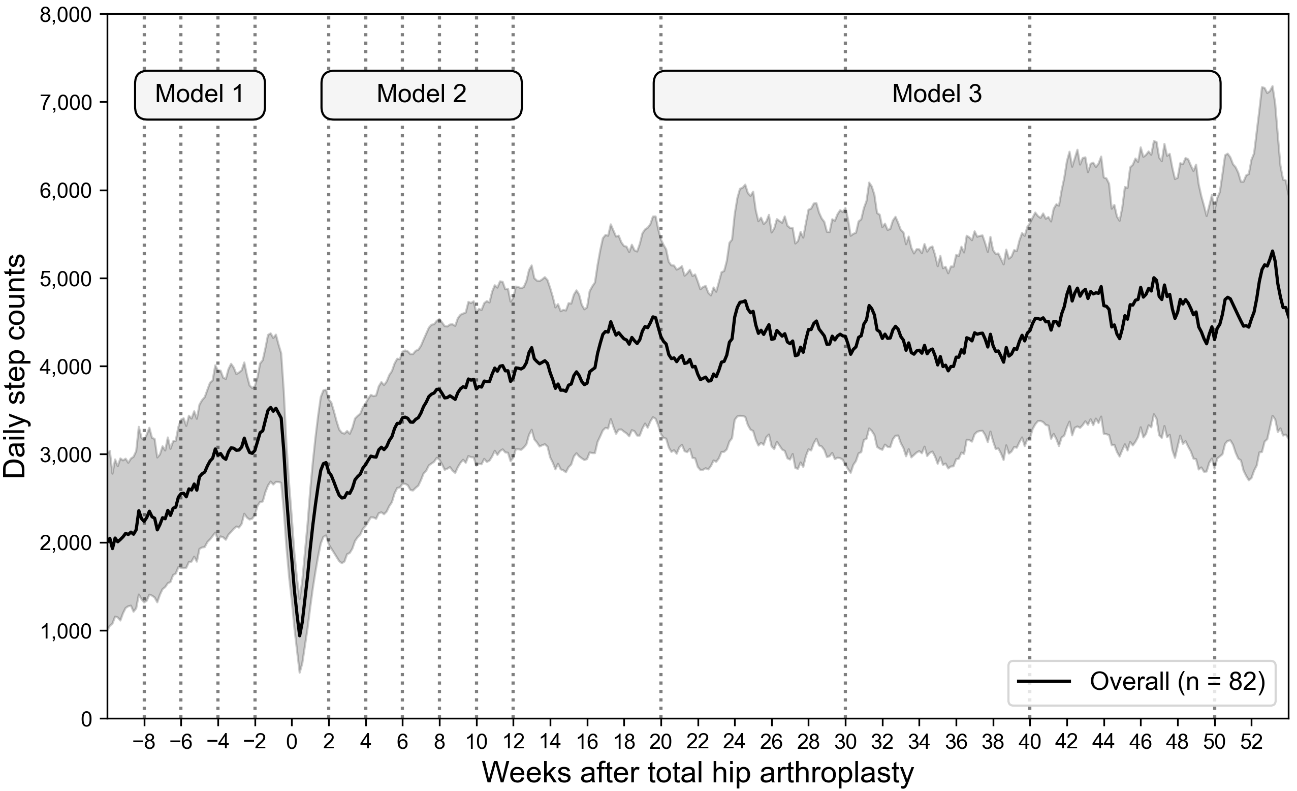


**S1 Fig**

Centered 7-day moving average of daily step counts across all patients. The band indicate 95% confidence intervals.
