# Supplementary material for: Using smartphone step counts to monitor patients with total hip arthroplasty: The impact of patients’ living arrangements and residential location
Source: PLoS One. 2025 Jun 27;20(6):e0326338. doi: 10.1371/journal.pone.0326338 (PMC12204548; doi:10.1371/journal.pone.0326338)
Supplement: S4 Fig — (DOCX) [file pone.0326338.s004.docx]

**
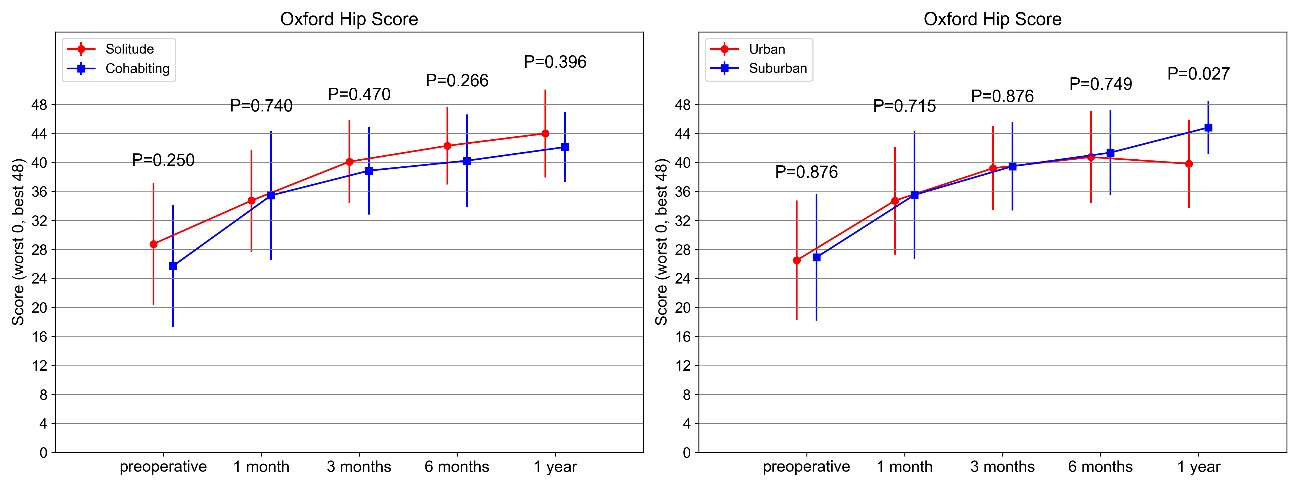
**

**S4 Fig**

Comparison of Hip Disability and Osteoarthritis Outcome Score (HOOS) by patients’ residential location. The error bars indicate the standard errors.
