# Supplementary material for: Using smartphone step counts to monitor patients with total hip arthroplasty: The impact of patients’ living arrangements and residential location
Source: PLoS One. 2025 Jun 27;20(6):e0326338. doi: 10.1371/journal.pone.0326338 (PMC12204548; doi:10.1371/journal.pone.0326338)
Supplement: S1 Table — (DOCX) [file pone.0326338.s005.docx]

**S1 Table.** Patient demographics of analyzed and excluded groups*

| Variables | Analyzed (n = 85) | Excluded (n = 148) | *P* value |
| --- | --- | --- | --- |
| Age at survey (*yr* [SD]) | 61.7 (9.4) | 66.4 (10.1) | < 0.001 |
| Sex (*no.* [%]) |  |  | 1.000 |
| Men | 14 (16) | 24 (16) |  |
| Women | 71 (84) | 124 (84) |  |
| BMI (*kg/m^2^* [SD]) | 24.8 (4.3) | 24.4 (4.6) |  |
| Diagnosis (*no.* [%]) |  |  | 0.399 |
| OA | 73 (86) | 135 (91) |  |
| ONFH | 5 (6) | 7 (5) |  |
| RA | 4 (5) | 2 (1) |  |
| SIF | 3 (4) | 4 (3) |  |
| ASA-PS (*no.* [%]) |  |  | 0.153 |
| I (normal, healthy) | 23 (27) | 27 (19) |  |
| II (mild systemic disease) | 57 (67) | 110 (78) |  |
| III (severe systemic disease) | 5 (6) | 4 (3) |  |
| Unknown | 0 | 7 |  |
| Living arrangements (*no.* [%]) |  |  | 1.00 |
| Solitude | 37 (44) | 64 (43) |  |
| Cohabiting | 48 (56) | 84 (57) |  |
| Residential location (*no.* [%]) |  |  | 0.497 |
| Urban | 44 (52) | 69 (47) |  |
| Suburban | 41 (48) | 79 (53) |  |

*SD, standard deviation; BMI, body mass index; OA, osteoarthritis; ONFH, osteonecrosis of the femoral head; RA, rheumatoid arthritis; SIF, subchondral insufficiency fracture; ASA-PS, American Society of Anesthesiologists physical status. †Significant at *P*<0.05.
