# Supplementary material for: Using smartphone step counts to monitor patients with total hip arthroplasty: The impact of patients’ living arrangements and residential location
Source: PLoS One. 2025 Jun 27;20(6):e0326338. doi: 10.1371/journal.pone.0326338 (PMC12204548; doi:10.1371/journal.pone.0326338)
Supplement: S2 Table — (DOCX) [file pone.0326338.s006.docx]

**S2 Table.** Comparison of mean daily step count at each time point*

| Variables | Overall  (n = 85) |  | Solitude  (n = 37) | Cohabiting  (n = 48) | *P* value |  | Urban  (n = 44) | Suburban  (n = 41) | *P* value |
| --- | --- | --- | --- | --- | --- | --- | --- | --- | --- |
| **Period of Model 1** |  |  |  |  |  |  |  |  |  |
| preoperative 8 weeks | 2,133 (1,988) |  | 2,699 (2,607) | 1,621 (1,008) | 0.104 |  | 2,127 (1,806) | 2,137 (2,168) | 0.987 |
| missing (n) | 45 |  | 18 | 27 |  |  | 26 | 19 |  |
| preoperative 6 weeks | 2,446 (2,213) |  | 2,972 (2,186) | 1,997 (2,175) | 0.122 |  | 2,859 (2,486) | 2,033 (1,861) | 0.190 |
| missing (n) | 35 |  | 14 | 21 |  |  | 19 | 16 |  |
| preoperative 4 weeks | 2,866 (2,494) |  | 3,242 (2,539) | 2,558 (2,452) | 0.296 |  | 3,365 (2,894) | 2,256 (1,762) | 0.073 |
| missing (n) | 25 |  | 10 | 15 |  |  | 11 | 14 |  |
| preoperative 2 weeks | 3,082 (2,192) |  | 3,960 (2,174) | 2,525 (2,038) | 0.010† |  | 3,910 (2,366) | 2,279 (1,683) | 0.002† |
| missing (n) | 18 |  | 11 | 7 |  |  | 11 | 7 |  |
|  |  |  |  |  |  |  |  |  |  |
| **Period of Model 2** |  |  |  |  |  |  |  |  |  |
| postoperative 2 weeks | 2,655 (2,838) |  | 3,336 (3,111) | 2,139 (2,534) | 0.103 |  | 3,280 (3,627) | 2,119 (1,815) | 0.120 |
| missing (n) | 20 |  | 9 | 11 |  |  | 14 | 6 |  |
| postoperative 4 weeks | 2,799 (2,430) |  | 3,614 (2,943) | 2,249 (1,858) | 0.039† |  | 3,251 (2,820) | 2,433 (2,029) | 0.188 |
| missing (n) | 18 |  | 10 | 8 |  |  | 14 | 4 |  |
| postoperative 6 weeks | 3,332 (2,490) |  | 4,177 (2,989) | 2,798 (1,979) | 0.053 |  | 4,127 (2,810) | 2,718 (2,049) | 0.033† |
| missing (n) | 23 |  | 13 | 10 |  |  | 17 | 6 |  |
| postoperative 8 weeks | 3,664 (2,423) |  | 4,677 (2,575) | 2,988 (2,089) | 0.010† |  | 4,191 (2,685) | 3,261 (2,155) | 0.155 |
| missing (n) | 25 |  | 13 | 12 |  |  | 18 | 7 |  |
| postoperative 10 weeks | 3,697 (2,550) |  | 4,712 (2,935) | 3,041 (2.054) | 0.026† |  | 4,318 (2,877) | 3,197 (2,171) | 0.114 |
| missing (n) | 29 |  | 15 | 14 |  |  | 19 | 10 |  |
| postoperative 12 weeks | 3,801 (2,678) |  | 4,739 (3,034) | 3,175 (2,247) | 0.046† |  | 4,197 (2,723) | 3,471 (2,640) | 0.323 |
| missing (n) | 30 |  | 15 | 15 |  |  | 19 | 11 |  |
|  |  |  |  |  |  |  |  |  |  |
| **Period of Model 3** |  |  |  |  |  |  |  |  |  |
| postoperative 20 weeks | 4,316 (2,914) |  | 5,345 (3,102) | 3,575 (2,585) | 0.057 |  | 4,513 (2,931) | 4,089 (2,953) | 0.640 |
| missing (n) | 42 |  | 19 | 23 |  |  | 21 | 21 |  |
| postoperative 30 weeks | 4,425 (3,924) |  | 6,660 (4,791) | 2,967 (2,380) | 0.012† |  | 4,068 (3,084) | 4,746 ‘4,610) | 0.595 |
| missing (n) | 47 |  | 22 | 25 |  |  | 26 | 21 |  |
| postoperative 40 weeks | 4,477 (2,874) |  | 5,639 (3,676) | 3,606 (1,719) | 0.062 |  | 4,785 (2,379) | 4,187 (3,318) | 0.543 |
| missing (n) | 50 |  |  |  |  |  | 27 | 23 |  |
| postoperative 50 weeks | 4,006 (2,939) |  | 5,397 (3,515) | 2,893 (1,796) | 0.017† |  | 4,463 (2,816) | 3,549 (3,068) | 0.358 |
| missing (n) | 49 |  | 21 | 28 |  |  | 26 | 23 |  |

*Mean value and standard deviation in parentheses. †Significant at *P*<0.05.
