# Supplementary material for: Using smartphone step counts to monitor patients with total hip arthroplasty: The impact of patients’ living arrangements and residential location
Source: PLoS One. 2025 Jun 27;20(6):e0326338. doi: 10.1371/journal.pone.0326338 (PMC12204548; doi:10.1371/journal.pone.0326338)
Supplement: S3 Table — (DOCX) [file pone.0326338.s007.docx]

**S3 Table.** Outcomes grouped by patients’ living arrangements and residential location*

| Variables | Overall  (n = 85) |  | Solitude  (n = 37) | Cohabiting  (n = 48) | *P* value |  | Urban  (n = 44) | Suburban  (n = 41) | *P* value |
| --- | --- | --- | --- | --- | --- | --- | --- | --- | --- |
| Postoperative days required to cane-walking independence (day [SD]) | 9.8 (8.7) |  | 8.4 (5.3) | 10.8 (10.6) | 0.175 |  | 9.2 (5.4) | 10.4 (11.3) | 0.521 |
| Length of stay (day [SD]) | 16.7 (8.1) |  | 15.1 (3.9) | 17.9 (10.1) | 0.080 |  | 16.1 (4.8) | 17.3 (10.6) | 0.501 |
| Discharge destination (n [%]) |  |  |  |  | >0.999 |  |  |  | 0.266 |
| Home | 52 (61) |  | 23 (63) | 29 (60) |  |  | 24 (55) | 28 (68) |  |
| Rehabilitation hospital | 33 (39) |  | 14 (38) | 19 (40) |  |  | 20 (45) | 13 (32) |  |
| Oxford Hip Score (Score [SD]) |  |  |  |  |  |  |  |  |  |
| Preoperative | 26.4 (8.7) |  | 28.5 (8.7) | 25.3 (8.7) | 0.250 |  | 26.1 (8.3) | 26.6 (9.1) | 0.876 |
| Missing data (n) | 42 |  | 22 | 20 |  |  | 27 | 15 |  |
| Postoperative 1 month | 35.0 (8.3) |  | 34.5 (7.1) | 35.4 (9.1) | 0.740 |  | 34.5 (7.5) | 35.5 (9.0) | 0.715 |
| Missing data (n) | 32 |  | 16 | 16 |  |  | 20 | 12 |  |
| Postoperative 3 months | 39.5 (5.6) |  | 40.1 (5.7) | 39.1 (5.7) | 0.470 |  | 39.2 (5.8) | 39.8 (5.7) | 0.876 |
| Missing data (n) | 37 |  | 17 | 20 |  |  | 24 | 13 |  |
| Postoperative day 6 months | 41.1 (6.0) |  | 42.3 (5.3) | 40.2 (6.4) | 0.266 |  | 40.7 (6.4) | 41.4 (5.8) | 0.749 |
| Missing data (n) | 43 |  | 20 | 23 |  |  | 25 | 18 |  |
| Postoperative 1 year | 42.9 (5.3) |  | 44.0 (6.1) | 42.1 (4.8) | 0.396 |  | 39.8 (6.1) | 44.8 (3.7) | 0.027† |
| Missing data (n) | 57 |  | 26 | 31 |  |  | 33 | 24 |  |
| HOOS Symptoms (Score [SD]) |  |  |  |  |  |  |  |  |  |
| Preoperative | 39.6 (23.7) |  | 41.8 (26.8) | 38.3 (22.0) | 0.561 |  | 36.8 (26.6) | 41.5 (21.7) | 0.678 |
| Missing data (n) | 39 |  | 20 | 19 |  |  | 25 | 14 |  |
| Postoperative 1 month | 70.5 (18.9) |  | 68.6 (24.6) | 71.8 (14.1) | 0.634 |  | 65.4 (22.4) | 74.5 (14.8) | 0.151 |
| Missing data (n) | 33 |  | 16 | 17 |  |  | 21 | 12 |  |
| Postoperative 3 months | 78.7 (15.9) |  | 80.8 (16.1) | 77.3 (17.9) | 0.426 |  | 73.4 (17.9) | 82.3 (13.5) | 0.077 |
| Missing data (n) | 38 |  | 18 | 20 |  |  | 25 | 13 |  |
| Postoperative 6 months | 79.0 (15.2) |  | 80.0 (14.6) | 78.3 (15.8) | 0.715 |  | 74.8 (12.5) | 82.6 (16.6) | 0.116 |
| Missing data (n) | 42 |  | 20 | 22 |  |  | 24 | 18 |  |
| Postoperative 1 year | 85.7 (13.5) |  | 87.1 (14.8) | 84.7 (12.9) | 0.658 |  | 78.2 (16.5) | 90.0 (9.4) | 0.054 |
| Missing data (n) | 55 |  | 25 | 30 |  |  | 33 | 22 |  |
| HOOS Pain (Score [SD]) |  |  |  |  |  |  |  |  |  |
| Preoperative | 51.2 (19.3) |  | 55.0 (16.4) | 49.0 (20.8) | 0.258 |  | 50.0 (21.9) | 52.0 (17.7) | 0.884 |
| Missing data (n) | 39 |  | 20 | 19 |  |  | 25 | 14 |  |
| Postoperative 1 month | 81.3 (14.8) |  | 81.8 (16.1) | 81.0 (14.1) | 0.798 |  | 78.5 (15.1) | 83.6 (14.4) | 0.313 |
| Missing data (n) | 33 |  | 16 | 17 |  |  | 21 | 12 |  |
| Postoperative 3 months | 84.3 (15.0) |  | 86.6 (14.3) | 82.7 (15.5) | 0.333 |  | 80.0 (17.3) | 87.1 (12.7) | 0.137 |
| Missing data (n) | 38 |  | 18 | 20 |  |  | 25 | 13 |  |
| Postoperative 6 months | 84.7 (13.2) |  | 88.1 (11.6) | 82.5 (14.0) | 0.163 |  | 83.1 (14.4) | 86.1 (12.2) | 0.526 |
| Missing data (n) | 42 |  | 20 | 22 |  |  | 24 | 18 |  |
| Postoperative 1 year | 89.8 (11.8) |  | 90.4 (13.4) | 89.4 (10.9) | 0.837 |  | 83.2 (15.3) | 93.7 (7.2) | 0.047 |
| Missing data (n) | 55 |  | 25 | 30 |  |  | 33 | 22 |  |
| HOOS ADL (Score [SD]) |  |  |  |  |  |  |  |  |  |
| Preoperative | 48.2 (19.5) |  | 51.3 (20.3) | 46.3 (19.2) | 0.432 |  | 47.5 (22.0) | 48.6 (18.1) | 0.962 |
| Missing data (n) | 39 |  | 20 | 19 |  |  | 25 | 14 |  |
| Postoperative 1 month | 73.0 (18.5) |  | 72.5 (21.0) | 73.4 (17.0) | 0.903 |  | 71.4 (19.1) | 74.3 (18.3) | 0.697 |
| Missing data (n) | 33 |  | 16 | 17 |  |  | 21 | 12 |  |
| Postoperative 3 months | 77.9 (16.6) |  | 80.8 (12.0) | 75.8 (19.1) | 0.269 |  | 74.9 (18.2) | 79.8 (15.5) | 0.368 |
| Missing data (n) | 38 |  | 18 | 20 |  |  | 25 | 13 |  |
| Postoperative 6 months | 83.2 (13.3) |  | 86.3 (10.5) | 81.2 (14.7) | 0.199 |  | 82.2 (13.0) | 84.0 (13.8) | 0.654 |
| Missing data (n) | 42 |  | 20 | 22 |  |  | 24 | 18 |  |
| Postoperative 1 year | 87.8 (13.6) |  | 91.9 (11.5) | 85.0 (14.5) | 0.157 |  | 82.2 (15.4) | 91.0 (11.8) | 0.122 |
| Missing data (n) | 55 |  | 25 | 30 |  |  | 33 | 22 |  |
| HOOS Sports (Score [SD]) |  |  |  |  |  |  |  |  |  |
| Preoperative | 51.4 (26.8) |  | 26.5 (24.0) | 26.1 (20.8) | 0.924 |  | 24.0 (22.9) | 27.8 (21.3) | 0.724 |
| Missing data (n) | 39 |  | 20 | 19 |  |  | 25 | 14 |  |
| Postoperative 1 month | 58.2 (24.7) |  | 53.3 (28.7) | 50.2 (25.8) | 0.736 |  | 48.4 (27.1) | 53.9 (26.8) | 0.476 |
| Missing data (n) | 33 |  | 16 | 17 |  |  | 21 | 12 |  |
| Postoperative 3 months | 67.2 (20.8) |  | 62.8 (24.2) | 55.1 (25.1) | 0.288 |  | 52.3 (27.7) | 62.3 (22.1) | 0.204 |
| Missing data (n) | 38 |  | 18 | 20 |  |  | 25 | 13 |  |
| Postoperative 6 months | 67.2 (20.8) |  | 70.2 (21.9) | 65.1 (20.3) | 0.450 |  | 63.4 (23.8) | 70.4 (17.8) | 0.293 |
| Missing data (n) | 42 |  | 20 | 22 |  |  | 24 | 18 |  |
| Postoperative 1 year | 73.3 (25.8) |  | 76.6 (31.4) | 71.2 (22.0) | 0.613 |  | 59.7 (34.8) | 81.3 (14.7) | 0.067 |
| Missing data (n) | 55 |  | 25 | 30 |  |  | 33 | 22 |  |
| HOOS QOL (Score [SD]) |  |  |  |  |  |  |  |  |  |
| Preoperative | 29.2 (17.0) |  | 30.9 (16.9) | 28.2 (17.3) | 0.666 |  | 28.6 (19.2) | 29.6 (15.7) | 0.888 |
| Missing data (n) | 39 |  | 20 | 19 |  |  | 25 | 14 |  |
| Postoperative 1 month | 58.8 (19.0) |  | 58.0 (23.1) | 59.3 (16.1) | 0.800 |  | 51.9 (20.3) | 64.2 (16.4) | 0.025† |
| Missing data (n) | 33 |  | 16 | 17 |  |  | 21 | 12 |  |
| Postoperative 3 months | 67.7 (17.3) |  | 68.8 (16.0) | 67.0 (18.3) | 0.681 |  | 59.2 (19.0) | 73.4 (13.5) | 0.010† |
| Missing data (n) | 38 |  | 18 | 20 |  |  | 25 | 13 |  |
| Postoperative 6 months | 70.4 (18.6) |  | 70.6 (19.9) | 70.2 (18.1) | 0.948 |  | 65.6 (20.3) | 74.5 (16.2) | 0.123 |
| Missing data (n) | 42 |  | 20 | 22 |  |  | 24 | 18 |  |
| Postoperative 1 year | 77.9 (20.7) |  | 83.3 (21.2) | 74.3 (20.2) | 0.256 |  | 68.2 (25.4) | 83.6 (15.6) | 0.088† |
| Missing data (n) | 55 |  | 25 | 30 |  |  | 33 | 22 |  |

*SD = standard deviation; HOOS = Hip disability and Osteoarthritis Outcome Score; ADL = activities of daily living; QOL = quality of life. †Significant at *P*<0.05.
