# Supplementary material for: Using smartphone step counts to monitor patients with total hip arthroplasty: The impact of patients’ living arrangements and residential location
Source: PLoS One. 2025 Jun 27;20(6):e0326338. doi: 10.1371/journal.pone.0326338 (PMC12204548; doi:10.1371/journal.pone.0326338)
Supplement: Attachment — Submitted filename: Response to Reviewers.docx [file pone.0326338.s008.docx]

**Journal Requirements:**

When submitting your revision, we need you to address these additional requirements.

1. Please ensure that your manuscript meets PLOS ONE's style requirements, including those for file naming. The PLOS ONE style templates can be found at

https://journals.plos.org/plosone/s/file?id=wjVg/PLOSOne_formatting_sample_main_body.pdf and

https://journals.plos.org/plosone/s/file?id=ba62/PLOSOne_formatting_sample_title_authors_affiliations.pdf

**Response:**

**We appreciate the information.**

**We have revised the manuscript according to the PLOS ONE's style requirements.**

2. In this instance it seems there may be acceptable restrictions in place that prevent the public sharing of your minimal data. However, in line with our goal of ensuring long-term data availability to all interested researchers, PLOS’ Data Policy states that authors cannot be the sole named individuals responsible for ensuring data access (http://journals.plos.org/plosone/s/data-availability#loc-acceptable-data-sharing-methods).

Data requests to a non-author institutional point of contact, such as a data access or ethics committee, helps guarantee long term stability and availability of data. Providing interested researchers with a durable point of contact ensures data will be accessible even if an author changes email addresses, institutions, or becomes unavailable to answer requests.

Before we proceed with your manuscript, please also provide non-author contact information (phone/email/hyperlink) for a data access committee, ethics committee, or other institutional body to which data requests may be sent. If no institutional body is available to respond to requests for your minimal data, please consider if there any institutional representatives who did not collaborate in the study, and are not listed as authors on the manuscript, who would be able to hold the data and respond to external requests for data access? If so, please provide their contact information (i.e., email address). Please also provide details on how you will ensure persistent or long-term data storage and availability.

**Response:**

**We have revised the Data Availability Statement to include the contact information of the Kyushu University Hospital Institutional Review Board instead of the corresponding author.**

**Text Changes (Data Availability Statement):**

Data cannot be shared publicly because of the need for consent from study participants. **Data are available from the Kyushu University Hospital Institutional Review Board (ijkseimei@jimu.kyushu-u.ac.jp) for researchers who meet the criteria for access to confidential data.**

3. Please include your full ethics statement in the ‘Methods’ section of your manuscript file. In your statement, please include the full name of the IRB or ethics committee who approved or waived your study, as well as whether or not you obtained informed written or verbal consent. If consent was waived for your study, please include this information in your statement as well.

**Response:**

**We have revised the ethics statement in Methods.**

**Text Changes (Materials and methods, Lines 74-79):**

This observational cohort study was approved by the **Kyushu University Hospital Institutional Review Board for Clinical Research (Registration number: 2021–197)**. It was conducted in accordance with the Strengthening the Reporting of Observational Studies in Epidemiology (STROBE) guidelines [11] and ethical standards of the Declaration of Helsinki. **Written informed consent was obtained from all participants prior to their inclusion in the study.**

4. Please review your reference list to ensure that it is complete and correct. If you have cited papers that have been retracted, please include the rationale for doing so in the manuscript text, or remove these references and replace them with relevant current references. Any changes to the reference list should be mentioned in the rebuttal letter that accompanies your revised manuscript. If you need to cite a retracted article, indicate the article’s retracted status in the References list and also include a citation and full reference for the retraction notice.

**Response:**

**We confirmed that there are no retracted papers in our References. There are no changes in References, only minor modifications to the format.**

**Response to Reviewer #1:**

Reviewer #1 (1): The research is quite satisfactory as regards methodology and scientific writing. The discussion and conclusions are very useful especially the remark by the authors that there is a breakdown in its traditional social structure and increasing social isolation in the older population in their community and its potential effect on results after hip replacement surgery

**Response:**

**We thank the Reviewer #1 for the positive comments. We thank Reviewer #1 for the positive comments. This study is the first to highlight the potential influence of living arrangements and residential location on perioperative physical activity in patients undergoing THA.**

**Response to Reviewer #2:**

Reviewer #2 (1): Introduction

Are there any studies that investigated the relation between residence and step count after THA. If not, please, mention that in the introduction as this adds to the novelty of your paper

**Response:**

**We are grateful to Reviewer #2 for critical comments and useful suggestions that helped us to improve our paper considerably. As suggested, we have revised the Introduction to clarify that this is the first study to investigate the association between residence and step count after THA.**

**Text Changes (Introduction, Lines 66-68):**

Therefore, living arrangements and the residential location could also be a crucial factor affecting perioperative activity in THA**; however, to the best of our knowledge, these associations have not been previously reported**.

Reviewer #2 (2):

Patients and methods

Did you use a validated version of HOOS and Oxford for the Japanese population.

**Response:**

**We thank Reviewer #2 for the valuable comment. Yes, we used validated Japanese versions of the HOOS and Oxford scores. We have revised the manuscript.**

**Text Changes (Materials and methods, Lines 123-125):**

For additional analysis, we collected the Oxford Hip Score (OHS) and Hip Disability and Osteoarthritis Outcome Score (HOOS), which are patient-reported outcome measures (PROMs) for hip osteoarthritis**, using the validated Japanese versions**.

Reviewer #2 (3):

Results

Patient flowchart is not clear. How many patients were excluded and why?

**Response:**

**We thank Reviewer #2 for the valuable comment.**

**We have clarified the patient flowchart in the manuscript, including the number of excluded patients and the reasons for exclusion.**

**Text Changes (Results, Lines 180-185):**

Overall, 233 patients were assessed for eligibility. **Among them, 60 did not have smartphones or were unable to install the application, leaving 173 patients (74.2%) with compatible smartphones. Of these, 54 declined to participate, and 119 wished to participate in the study. Among the 119 patients, data from 34 were excluded due to incomplete or invalid step-count data. Finally, data from 85 patients who underwent THA and had valid step-count data were included in the analysis** (Fig 2)**.**

Reviewer #2 (4):

Discussion

Could you elaborate more on why patients living in Solitude had more step count than cohabiting patients?

**Response:**

**We thank the Reviewer #2 for the valuable comment. We have revised the text.**

**Text Changes (Discussion, Lines 274-278):**

**Our study found that solitary patients exhibited significantly higher step counts than cohabiting patients during the plateau phase of postoperative recovery, which spanned from postoperative week 20 to week 50, suggesting that living alone may require greater physical autonomy in daily life. This finding may provide helpful information for surgical decision-making.**

Reviewer #2 (5):

Was there any relation between PROMs and step count?

**Response:**

**We thank the Reviewer #2 for the valuable comment. We have revised the text.**

**Text Changes (Discussion, Lines 265-267):**

Solitude patients maintained higher activity levels from the preoperative to the postoperative period than cohabiting patients did**, a difference that was not captured by OHS or HOOS**.
